# How specific molecules can lead to overeating

**DOI:** 10.7554/eLife.93090

**Published:** 2023-10-27

**Authors:** María Gabriela Blanco, Diego Rayes

**Affiliations:** 1 Instituto de Investigaciones Bioquímicas de Bahía Blanca (INIBIBB), UNS–CONICET Bahía Blanca Argentina; 2 Departamento de Biología, Bioquímica y Farmacia, Universidad Nacional Del Sur Bahía Blanca Argentina

**Keywords:** feeding, advanced glycation end-products, glod-4, elt-3, tyramine, pharyngeal pumping, *C. elegans*

## Abstract

A molecular pathway involving compounds found in processed foods and biogenic amines increases food intake and aging in the roundworm *C. elegans*.

**Related research article** Muthaiyan Shanmugam M, Chaudhuri J, Sellegounder D, Sahu AK, Guha S, Chamoli M, Hodge B, Bose N, Amber C, Farrera DO, Lithgow G, Sarpong R, Galligan JJ, Kapahi P. 2023. Methylglyoxal-derived hydroimidazolone, MG-H1, increases food intake by altering tyramine signaling via the GATA transcription factor ELT-3 in *Caenorhabditis elegans*. *eLife* **12**:e82446. doi: 10.7554/eLife.82446.

Toasted marshmallows, roasted nuts and fried potatoes are just a few examples of foods that owe their unique taste to a chemical reaction that produces molecules called advanced glycation end-products. These compounds form when sugars react spontaneously with proteins or lipids without the involvement of enzymes. Modern processed foods, especially those subjected to high temperatures for long periods of time – as happens with grilling, roasting or frying – are particularly high in advanced glycation end-products (Liu et al., 2020), but these compounds can also be produced in the body during metabolic processes (Reddy et al., 2022).

Advanced glycation end-products (AGEs) have been linked to several medical conditions, particularly diabetes, where they contribute to tissue damage and inflammation (Ramasamy et al., 2005). More recent research suggests that they can also increase neuronal damage and reduce the life span of roundworms (Chaudhuri et al., 2016). Now, in eLife, Pankaj Kapahi and colleagues based at various research institutes in the United States – including Muniesh Muthaiyan Shanmugam (Buck Institute for Research on Aging) as first author – report on the intriguing connection between diet, molecular signaling, feeding behavior and neurodegeneration in the roundworm *C. elegans* (Muthaiyan Shanmugam et al., 2023).

Shanmugam et al. compared the food intake of healthy worms and knock-out worms that lacked an enzyme called GLOD-4, which is responsible for degrading a compound known as methylglyoxal, a precursor of many AGE compounds (Chaudhuri et al., 2016). The high level of AGE compounds in the knock-out worms appeared to increase their food intake (as indicated by a high pharyngeal pumping rate). Consuming the AGE compound hydroimidazolone also caused the healthy worms to eat more. This suggests that the relationship between the accumulation of AGE compounds and over-eating could be conserved across the animal kingdom.

The experiments also showed that the response to AGE compounds is gradual, not immediate, with a pronounced increase in food intake occurring 24 hours after the introduction of hydroimidazolone. This temporal dimension makes it more difficult to understand how dietary choices impact feeding behavior over time. RNA sequencing also revealed that genes that code for neurotransmitters, and genes that code for proteins regulating feeding behavior, were upregulated in the knock-out worms.

Previous research has shown that biogenic amines – biomolecules that contain one or more amine groups – play a role in feeding behavior throughout the animal kingdom (Greer et al., 2008). Given this, and the differential expression of genes observed in the knock-out worms, Shanmugam et al. investigated how mutations in genes related to the synthesis of amines affected food intake in worms exposed to hydroimidazolone. They found that mutations in an enzyme called TDC-1 – which is responsible for synthesizing the amine tyramine, the invertebrate equivalent of adrenaline – stopped the overfeeding that is usually induced by hydroimidazolone.

Moreover, *glod-4* mutant animals without tyramine had decreased damage in their nervous system and longer lifespans. This suggests that tyramine is a key regulator of overeating induced by hydroimidazolone, but more research is needed to fully understand the role of this particular amine. The negative effects of AGE compounds in the knock-out worms could be due to excessive eating triggered by tyramine, or it might be that tyramine itself is causing damage, as noted in a previous study (De Rosa et al., 2019). Additionally, two G-protein-coupled receptors that respond to tyramine were also implicated in the process.

The study also unravels a critical aspect of how AGE compounds influence feeding behavior at a molecular level. Specifically, exposure to hydroimidazolone increases the transcription of the gene that codes for the enzyme TDC-1, and this upregulation relies on the presence of a transcription factor called ELT-3 (Figure 1). This finding enriches our understanding of the mechanisms through which AGE compounds impact feeding behavior.

**Figure 1.**
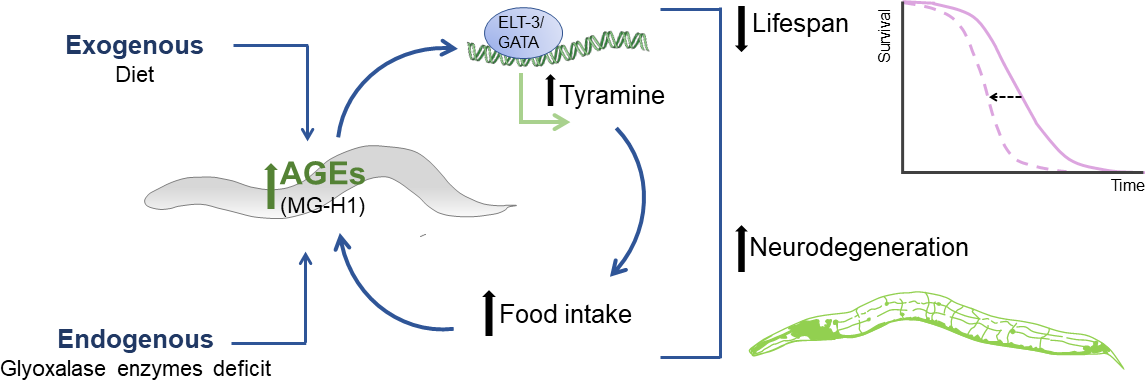
The relationship between AGE compounds and food intake. Advanced glycation end-products (AGEs) can accumulate due to dietary intake (top left) or due to a lack of enzymes that break down their precursors (bottom left). This accumulation leads to the build-up of an AGE compound called hydroimidazolone (MG-H1), which stimulates the overexpression of tyramine through the ELT-2/GATA transcription factor pathway (top middle). The upregulation of tyramine promotes increased food intake, which, in turn, further increases the amount of hydroimidazolone in the body. This positive feedback loop, mediated by AGE compounds, ultimately reduces the lifespan of *C. elegans* (top right), and also accelerates neurodegeneration (bottom right).

It is becoming increasingly evident that AGE compounds actively participate in the regulation of physiological functions, rather than being passive byproducts of metabolic processes. The work of Shanmugam et al. demonstrates that, in *C. elegans* at least, overeating due to an accumulation of AGE compounds depends on the modulation of biogenic amine signals. This knowledge could contribute to a better understanding of a range of systemic diseases, including diabetes, obesity and various neurodegenerative disorders.

Shanmugam et al. also highlight the evolutionary importance of these processes. The changes to tyramine signaling responsible for the detrimental effects of AGE compounds in *C. elegans* raise the tantalizing possibility that aminergic modulation may have similar effects in humans. For example, prolonged adrenaline release in humans due to chronic stress has been associated with obesity, diabetes and premature aging (Yiallouris et al., 2019).

The work of Shanmugam et al. offers valuable insights into the interplay between dietary choices, molecular signaling and health. By unveiling a new pathway involving AGE compounds and aminergic signaling in *C. elegans*, this research enriches our understanding of the regulation of feeding behavior. It also encourages a re-evaluation of the impact of dietary AGE compounds on human health and motivates further exploration of their potential role in the development of chronic diseases.
